# Effects of orofacial myofunctional therapy on masticatory function in individuals submitted to orthognathic surgery: a randomized trial

**DOI:** 10.1590/1678-7757-2017-0164

**Published:** 2018-01-16

**Authors:** Daniela Galvão de Almeida Prado, Giédre Berretin-Felix, Renata Resina Migliorucci, Mariana da Rocha Salles Bueno, Raquel Rodrigues Rosa, Marcela Polizel, Isadora Ferraz Teixeira, Maria Beatriz Duarte Gavião

**Affiliations:** 1 Universidade Estadual de Campinas, Faculdade de Odontologia de Piracicaba, Departamento de Ciências Fisiológicas, Piracicaba, SP, Brasil; 2 Universidade de São Paulo, Faculdade de Odontologia de Bauru, Departamento de Fonoaudiologia, Bauru, SP, Brasil; 3 Clínica particular, Bauru, SP, Brasil; 4 Clínica particular, Piracicaba, SP, Brasil; 5 Universidade Estadual de Campinas, Faculdade de Odontologia de Piracicaba, Piracicaba, SP, Brasil; 6 Universidade Estadual de Campinas, Faculdade de Odontologia de Piracicaba, Departamento de Odontologia Infantil, Piracicaba, SP, Brasil

**Keywords:** Dentofacial deformities, Orthognathic surgery, Myofunctional therapy, Mastication, Electromyography

## Abstract

**Objectives:**

The esthetic and functional results of orthognathic surgery of severe dentofacial deformities are predictable, however there are differences regarding the effects on stomatognathic system. The aim was to investigate the effects of orofacial myofunctional therapy (OMT) on the masticatory function in individuals with dentofacial deformity submitted to orthognathic surgery (OGS).

**Material and Methods:**

Forty-eight individuals (18-40 years) were evaluated, 14 undergoing OMT (treated group-TG), 10 without this treatment (untreated group-UTG) and 24 in a control group with normal occlusion; for clinical aspects the data of an individual was missed (n=46). Chewing was performed using the Expanded protocol of orofacial myofunctional evaluation with scores (OMES-E). Muscle tone and mobility were also analyzed before (P0), three (P1) and six months (P2) after OGS. Surface electromyography of the masseter and temporalis muscles was performed, considering the parameters amplitude and duration of act and cycle, and the number of masticatory cycles. The OMT consisted of ten therapeutic sessions along the postoperative period. The results were compared using parametric and non-parametric tests.

**Results:**

TG showed higher scores in P1 and P2 than P0; for the masticatory type the scores in P2 were significantly higher than P0. In addition, the proportion of individuals with adequate tone of lower lip and adequate tongue mobility for TG increased significantly from P1 and P2 in relation to P0. The EMG results showed a decrease in act and cycle duration in P2 in relation to P0 and P1 for the TG; furthermore the values were close to controls. An increase in the number of cycles from P0 to P2 was also observed, indicating faster chewing, which may be attributed to an improvement of balanced occlusion associated with OMT.

**Conclusion:**

There were positive effects of OMT on the clinical and electromyography aspects of chewing in individual submitted to orthognathic surgery.

## Introduction

Individuals with severe dentofacial deformities (DFD) submitted to orthodontic treatment and orthognathic surgery (OGS) usually are seeking improvements in facial esthetics and function of the stomatognathic system; consequently, better occlusal relations can be achieved^4^. The esthetic and functional results are predictable, but there are differences regarding the respective effects^23^.

Chewing is an important function of the stomatognathic system; the ideal pattern is bilaterally alternated, with sealed lips and jaw rotation movements with no movement of the head or other body parts, enabling the distribution of masticatory forces with functional and muscular balance, but depending on factors of occlusal balance^25^.

Chewing can be altered in individuals with DFD^2^. In Class III malocclusion the vertical mandibular movements are predominant, with utilization of the tongue dorsum to crush the food against the palate and little or no action of the buccinator muscles. In Class II malocclusion, usually, the lack of lip sealing can be observed in the presence of long face, determining little use of orbicularis oris muscles and buccinators, accompanied by less movement of tongue lateralization^14^ ^,^ ^24^.

Some protocols for clinical evaluation of chewing have been developed in the area of Orofacial Myology, such as the Expanded protocol of orofacial myofunctional evaluation with scores (OMES-E)^7^ ^,^ ^8^, which has been proved to be a valid and reliable instrument for orofacial myofunctional evaluation, allowing grading of the respective conditions within the limits of selected items^7^. This protocol comprises analysis of the posture of components of the stomatognathic system; mobility of lips, tongue, jaw and cheeks and evaluation of orofacial functions, for which scores were assigned according to the severity of change.

An instrumental method to evaluate masticatory function consists in the surface electromyography (EMG electromyography), which records muscle activity in microvolts (μV) and in seconds, through bipolar electrodes. The EMG detects the electric potential of the muscle fibers and can simultaneously record the muscles of the craniomandibular region in both sides. EMG records can provide information about muscle function in experimental conditions^3^.

Most studies about masticatory function in individuals with DFD submitted to orthodontic-surgical treatment showed that the EMG of masticatory muscles is lower compared to subjects with normal occlusion^16^ ^,^ ^27^. Moreover, changes in masticatory function or in its components after correction of DFD by OGS are evident. The period of time for occurrence of changes is controversial and may be related to differences in evaluation methods and treatment types^21^.

Regarding the duration of chewing, Ueki, et al.^26^ (2009) found no changes in this characteristic after OGS in Class III malocclusion, and the same was found by Youssef, et al.^28^ (1997) in individuals with Class II and III malocclusion. Conversely, a reduction was observed in the duration of muscle activity in the postoperative period compared to the preoperative in patients with Class III malocclusion^15^. It is relevant to consider the methodological differences between researches, since the knowledge about adaptation of this function with the correction of form still has limitations.

A recent research showed increasing trend of the total number of chewing cycles after 36 months of orthodontic-surgical treatment in patients with Class III malocclusion, determining improvement in the balance of the masticatory muscles after surgery^19^.

Nevertheless, the literature about orofacial myofunctional therapy (OMT) for patients submitted to OGS has been controversial, probably due to methodological differences^15^ ^,^ ^17^ ^,^ ^22^. Due to alterations of the orofacial structures in individuals with DFD after OGS, a new proprioceptive scheme must be acquired so the soft structures may satisfactorily perform their functions. Therefore, to complement clinical evaluation and to understand the functional changes in DFD, it is important to study the effect of OMT on the functional aspects of masticatory muscles before and after surgical correction of DFD, to elucidate the adaptation of these muscles after surgery.

In this context, the efficacy of OMT rehabilitation in a short time must be more precisely investigated to know if the functionality of the stomatognathic system and the possible relapses caused by inadequate maintenance of adaptive patterns could be recovered early^15^.

Thus, the aim of this study was to determine the effects of OMT on the clinical and electromyography aspects of masticatory function in individuals with DFD, before, three and six months after OGS.

## Material and methods

The study was approved by the Institutional Review Board under protocol 074/2012. The registration number of clinical trial is RBR-4mt6yr.

### Sample selection

The study is a randomized longitudinal clinical trial, parallel with allocation ratio of 1:1. Young adults with DFD, receiving orthodontic treatment before OGS and attending the Maxillofacial Surgery area of the University were enrolled, forming the experimental group. Furthermore, a control group without DFD was obtained, age- and gender-matched with the individuals undergoing treatment. All individuals signed a free informed consent form. The procedures were carried out along 2013 to 2015.

The sample was selected by convenience. The inclusion criteria of the experimental group were healthy individuals, aged from 18 to 45 years, both genders, presenting at least 24 teeth, with skeletal Class II or III malocclusion, diagnosed by cephalometric radiographs and clinical evaluation carried out before OGS by the staff of the Maxillofacial Surgery Area. The control group should present good relation between dental arches; overbite and overjet ranging from 1 to 3 mm; all natural teeth at least up to the second molar; nasal breathing; the face height should be similar to the face width to be classified into medium facial type, evaluated using a digital caliper (Mitutoyo, Santo Amaro-SP, Brazil).

Exclusion criteria for both groups were neurological, psychiatric or intellectual deficits, partially or totally edentulous patients and the presence of cleft lip or palate. The respective information was obtained by interview and clinical evaluation.

**Figure 1:**
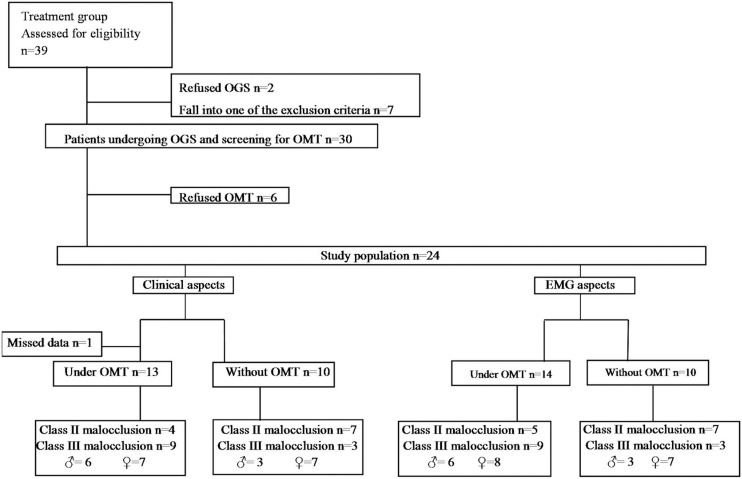
Flow chart: sample distribution according to groups and period of evaluation

After OGS, the experimental group was composed of 24 individuals allocated in two sub-groups, namely those who received OMT (Treated group - TG) and those without OMT (untreated group - UTG) (Figure 1). The allocation was performed by randomization. The numbers 1-24 were randomized on an Excel worksheet, and the first 14 numbers drawn were part of the TG and the last 10 of the UTG. In evaluations of clinical aspects, data of one individual of TG were missed between the second and third evaluations, who was excluded from the analysis.

The final sample of clinical aspects was composed of 13 individuals (29.31±8.87 years) allocated in TG and 10 in the UTG (31.20±7.02 years), both with their corresponding controls (mean age 28.39±7.34 years and mean age 28.10±5.30 years), respectively. For EMG aspects, 14 individuals (29.62±8.78 years) allocated in TG and 10 in the UTG (31.20±7.02 years), both with their corresponding controls (mean age 28.38±X years and mean age 28.10±5.30 years), respectively.

After the last evaluation, OMT was offered to the UTG.

Below, the sample characteristics according to the malocclusion and surgery:

– Class II - Sagittal osteotomy of the mandibular ramus (TG n=1; UTG n=7) and sagittal osteotomy of the mandibular ramus with maxilla setback (TG n = 3; UTG n=0);
– Class III - Le Fort I osteotomy (TG n=4; UTG n = 1); Le Fort I osteotomy and mandibular setback (TG n=5; UTG n = 1); Mandibular setback (TG n=0; UTG n=1).

One individual with class II malocclusion was excluded from the clinical analyses due to the missed data, but included on the instrumental analyses.

Individuals with Class II and III malocclusion were compared by the t test or Mann Whitney test for all variables according to data normality. Since no significant difference was found, the data were pooled.

TG and UTG were evaluated in three stages: before, one or two weeks before OGS; and post stages, three and six months after OGS. The OMT was applied in the postoperative period, 30 days after surgery, with 10 sessions, one per week. The control group was evaluated in a single period.

### Procedures

#### Clinical evaluation of chewing

The masticatory function was evaluated using OMES-E^8^, considering that the higher the score, the better the function. The study analyzed the incision, masticatory type, movements of the head or other body parts, altered head posture and food escape. These assessments were recorded using a Coolpix L810 camera (Nikon, São Paulo, SP, Brazil). Three examiners, professional experts in the area, performed the analysis; the agreement between at least two of them was taken into account, according to the assigned scores.

Following the protocol, mastication was recorded with the individual sitting in a chair with a backrest, the feet resting on the floor at a standardized distance (1 m) from the camera lens, which was mounted on a tripod with focus on the face, neck and shoulders. The individuals chewed one wafer biscuit and in their habitual manner.

The bite was evaluated during filming and the scores were attributed as following: 1=when the individual did not bite the food but broke it into pieces with his hands before bringing it to his mouth; 2=biting with the molars; 3 = biting with the canines and the premolars; 4=biting with the incisors.

The counting of masticatory strokes for mastication type was made considering the jaw movements of opening and closing until occurrence of contact of teeth. The following scores were attributed: 1=when the patient did not perform the function; 2=when the masticatory strokes occurred on the same side 78-94% of the times; 3 = chronic unilateral, when the masticatory strokes occurred on the same side 95-100% of the time, or anterior when occurred in the region of the incisors and canines; 4=unilateral preference grade 2 when the masticatory strokes occurred on the same side 78-94% of the times; 6=unilateral preference grade 1 when the masticatory strokes occurred on the same side 61-77% of the times; 8=simultaneously bilateral, with the masticatory strokes occurring on both sides of the oral cavity 95% of the times; 10=when it was bilateral and alternate, i.e., the masticatory strokes occurred on each side 50% of the times, or 40% on one side and 60% on the other.

In addition, it was analyzed the movement and/or altered posture of the head and of other parts of the body, food escape and uncoordinated jaw movements. Score 1 was attributed to the presence of the alteration and score 2 to the absence.

#### Clinical evaluation of tone and mobility

During clinical evaluation, the mobility of the lips and tongue was observed, and the individuals were asked to perform the following movements: Lips: protrude closed, retract closed, protrude open, retract open, protrude closed to the right, protrude closed to the left, pop protracted, pop retracted. Tongue: protrude and retract, touch right and left commissures and upper and lower lips sequentially, touch incisive papilla, touch right cheek, touch left cheek, click tip, suck tongue on palate. If the individual did not perform one of the tasks, the mobility was considered altered. The tone of the upper and lower lip was evaluated and classified as normal, reduced or increased; both reduced and increased were considered as altered.

#### Instrumental examination

Data were collected at the Ultrasonography and Electromyography Laboratory of the Pediatric Dentistry Department (FOP-UNICAMP), which has proper environment and conditions for adequate collection of EMG signal. EMG recordings were obtained from four channels of the electromyography (EMG SYSTEM, São José dos Campos-SP, Brazil), model 810c. According to the manufacturer's recommendation the calibration used was −2500 to +2500 μV. The instrument was connected to a computer for data storage and subsequent analysis.

The evaluations were performed with the individual sitting on a chair; the surface of the skin over the muscles was cleaned with alcohol wipes (70th GL) in order to remove the superficial fat, dead cells, reduce the skin impedance and thus avoid interference and ensure signal quality. The muscles evaluated were: right masseter (RM), left masseter (LM), right temporalis (RT) and left temporalis (LT).

Disposable surface double Hal electrodes were used (Miotec Biomedical Equipment, Porto Alegre-RS, Brazil), placed on the skin with conductive paste and fixated using micropore^®^. The electrodes were placed on the belly of the masseter and anterior temporalis as follows: masseter - between the level of the zygomatic arch and gonial angle, close to the occlusal plane level; anterior temporalis muscle - in front of the hairline, in the longitudinal direction of the anterior bundle fibers defined by palpation during clenching. The ground electrode was fixated on the right wrist of the patient after application of conductive paste.

Mastication of a latex rubber with 2.0-cm length and 1.0-cm diameter was carried out for 60 seconds in the usual manner. In addition, the maximum isometric voluntary contraction (MIVC) was performed along 20 seconds; the subject was instructed to bite with maximum possible force (teeth clenching) for three times and the mean of the respective records was considered for analysis. The results were obtained in μV Root Mean Square (RMS), which gives the number of motor units activated (recruitment) or the amplitude of the EMG signal. During analysis of the electromyograms, the first two seconds were discarded and 10 subsequent seconds were considered.

The percentage of muscle activity was calculated as follows: (RMSx100)/MIVC. Additionally, the duration of chewing act and cycle in seconds were obtained. The masticatory act is the amount of time that the muscle remains active during the occlusal phase. The chewing cycle involves three phases, namely opening, closing and occlusal phase (Figure 3).

### Analyses of chewing side preference

Furthermore, the chewing side preference was evaluated to better understand the variations on EMG records along time. The respective task was video recorded (Nikon Coolpix L810, São Paulo-SP, Brazil). The subject remained seated on a chair with a backrest, with their feet resting on the floor at a standardized distance (1 m) from the camera lens, which was mounted on a tripod with focus on the face, neck and shoulders. The subjects chewed one wafer biscuit as usual. Analysis of the video and classification of the preferred side was performed by three expert examiners in the area; the agreement between at least two of them was taken into account.

### Orofacial myofunctional therapy

In the preoperative period, after completion of clinical assessment, the patient received orientation and clarification for the orofacial myofunctional conditions resulting from the DFD and myofunctional consequences arising from OGS. Guidelines were reported about surgical trauma, facial edema, decreased sensitivity and facial movements, diet, oral hygiene and postoperative care.

**Figure 2:**
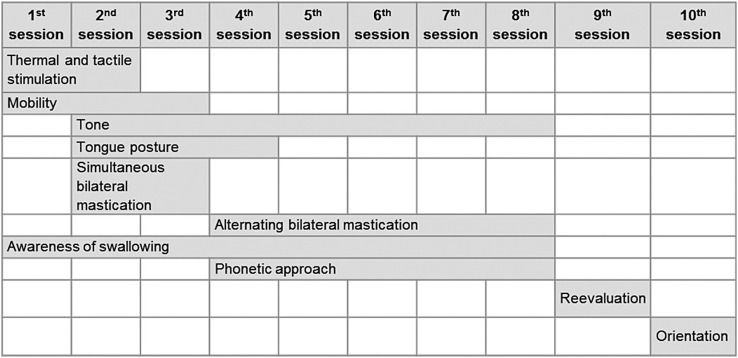
Aspects addressed during therapeutic sessions

In the treatment process, the “Post Orthognathic surgery therapy Protocol” was applied, which was prepared by the project team based on the literature and effective application in 11 individuals (unpublished data). The protocol consists of 10 sessions, one *per* week, starting 30 days after OGS and addressing the sensitivity, tone, mobility, adequacy of posture of lips and tongue, training and adequacy of orofacial myofunctional functions. Figure 2 shows the aspects addressed on each session.

**Figure 3:**
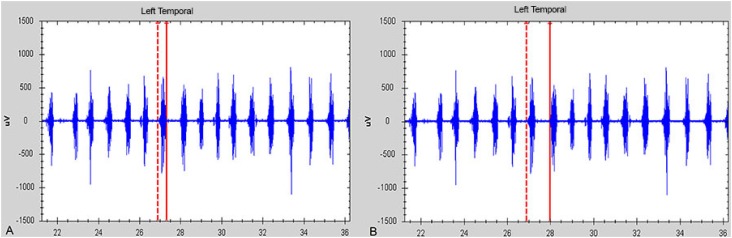
(a) Electromyography diagram showing the chewing act, (b) Electromyography diagram showing the chewing cycle

### Statistical analysis

Intra-subgroup comparisons (TG and UTG) before, three and six months after surgery were carried out, using ANOVA and *post hoc* Tukey test or Friedman and *post hoc* Dunn test, according to data distribution. The comparison between subgroups with their controls was performed using Kruskal-Wallis and *post hoc* Dunn test for data with scores, and Anova with *post hoc* Dunnet test for numeric data. The Fisher's exact test was used to compare frequencies. A significance level of 5% was adopted.

## Results

The values of the maximum score of OMES-E protocol^8^ for TG and UTG in each evaluation period are shown in Table 1. Significant increase was observed in TG from P0 to P1 and P0 to P2. The respective differences were not observed in UTG. Both groups showed significantly lower total scores than their controls in all periods.

**Table 1:** Mean values (standard deviation) of OMES-E scores in each period for the treated group (TG), untreated group (UTG) and control group (CG)

	Maximum score of OMES-E	
	TG	UTG
P0	13.23	15
	*±3.06	±3.19
P1	15,92*	15,4
	*±3.84	±366*
P2	16*	16,2*
	±3.51*	±3.67*
GC	19,62	19,3
	±0.65	±1.34

* p<0.05 statistically significant

Legend: P0: before surgery; P1: 3 months after surgery; P2: 6 months after surgery

TG: treated group; UTG: untreated group; CG: control group Statistical tests used: Comparison between periods: Anova/Tukey; Comparison with control: Kruskal-Wallis/Dunn

The score values for “bite” and “masticatory type” are in Table 2. No significance differences for “bite” were found between periods. For “masticatory type”, TG scores in P2 were significantly higher than in P0. In P0 and P1 the TG and UTG showed lower scores than the CG, whereas in P2 only TG showed lower scores than the CG.

**Table 2:** Mean values (±standard deviation) of the OMES-E protocol items according to period of evaluation for the TG, UTG and CG

		Bite# (maximum score: 4)		Masticatory type# (maximum score: 10)			Movements of the head+		Altered head posture+		Food escape+	
		TG	UTG	TG	UTG		TG	UTG	TG	UTG	TG	UTG
							n (%)	n (%)	n (%)	n (%)	n (%)	n (%)
	Mean±SD	3.61	3.9	4.3	6.20	Presence	4 (31)	2 (20)	5 (38)	8 (80)	0 (0)	1 (10)
P0		±0.87	±0.31	±2.56	±3.3	Absence	9 (69)	8 (80)	8 (62)	2 (20)	13 (100)	9 (90)
	Median	4.00	4.00	4.00	6.00							
	Mean±SD	3.77	4.00	*6.30	6.40	Presence	1 (8)	4 (40)	1 (8)	6 (60)	0 (0)	0 (0)
P1		±0.83	±0.00	±3.04	±3.09	Absence	12 (92)	6 (60)	12(92)	4 (40)	13 (100)	10 (100)
	Median	4.00	4.00	6.00	*6.00*		*				*	
	Mean±SD	3.69	3.70	6.92*	7.60*	Presence	3 (23)	3 (30)*	5 (38)	7 (70)	0 (0)	1 (10)
P2		±0.86	±0.95	±2.78	±2.63	Absence	10 (77)	7 (70)	8 (62)	3 (30)	13 (100)	9 (90)
	Median	4.00	4.00	8.00*	8.00					*		
	Mean±SD	3.92	3.90	10.00	9.60	Presence	0 (0)	0 (0)	4 (31)	2 (20)	0 (0)	0 (0)
CG		±0.27	±0.32	±0.00	±1.3	Absence	13 (100)	10 (100)	9 (69)	8 (80)	13 (100)	10 (100)
	Median	4.00	4.00	10.00	10.00							

* p<0.05 statistically significant

Legend: P0: before surgery; P1: 3 months after surgery; P2: 6 months after surgery TG: treated group; UTG: untreated group; CG: control group

The alterations of head movements and posture, as well as food escape, were recorded as present or absent (Table 2). Thus, the respective frequencies are demonstrated, and most individuals of TG showed absence of alterations in head movements in all evaluations, as well as for food escape; only one individual presented food escape in P0 and P2. Both control groups presented no alteration in those two items of OMES, as expected. Nevertheless, for head posture, the experimental and control groups presented from 2 to 8 individuals with alterations along the evaluations.

Table 3 presents the frequency of individuals with altered muscle tone. At P0, TG and UTG showed higher proportions of individuals with altered tone for upper and lower lips and tongue. At P1 the respective differences were observed for lower lip and tongue, whereas at P2 the proportion of individuals was higher for lower lip in UTG and for tongue in TG compared with their controls. Moreover, the proportions of individuals with adequate tone of lower lip for TG increased significantly from P0 compared with P1 and P2. No significant differences for lip mobility occurred between periods. The TG presented fewer individuals with alteration in tongue mobility in P1 and P2 than in P0. In P1 and P2, only UTG showed more individuals with alteration than CG (Table 3).

**Table 3:** Frequency of individuals according to muscle tone and mobility in each period of evaluation for the TG, UTG and CG

			Tone					Mobility			
		Upper lip		Lower lip		Tongue		Lips		Tongue	
		TG	UTG	TG	UTG	TG	UTG	TG	UTG	TG	UTG
		n (%)	n (%)	n (%)	n (%)	n (%)	n (%)	n (%)	n (%)	n (%)	n (%)
P0	adequate	7 (53.84)	4 (40.00)	0 (0.00)	1(10.00)	1 (7.69)	1 (10.00)	7 (53.84)	3 (30.00)	4 (30.77)	3 (30.00)
	alteration	6 (46.15)	6 (60.00)	13 (100)	9 (90.00)	12 (92.31)	9 (90.00)	6 (46.15)	7 (70.00)	9 (69.23)	7 (70.00)
P1	adequate	9 (69.23)	6 (60.00)**	**6 (46.15)	4 (40.00)	6 (46.15)	4 (40.00)	10 (76.92)	3 (30.00)**	**11 (84.61)	1 (10.00)
	alteration	4 (30.77)*	4 (40.00)*	7 (53.84)	6 (60.00)**	7 (53.84)**	6 (60.00)**	3 (23.07)**	7 (70.00)	2 (15.38)	9 (90.00)**
P2	adequate	0 (53.80)	8 (80.00)	8 (61.54)*	4 (40.00)*	6 (46.15)*	5 (50.00)*	11 (84.61)	4 (40.00)	11 (84.61)	1 (10.00)
	alteration	3 (46.15)	2 (20.00)	5 (38.46)	6 (60.00)*	7 (53.84)*	5 (50.00)	2 (15.38)	6 (60.00)	2 (15.38)	9 (90.00)**
CG	adequate	12 (92.30)	9 (90.00)	11 (84.61)	9 (90.00)	13 (100.00)	9 (90.00)	11 (84.61)	7 (70.00)	8 (61.54)	7 (70.00)
	alteration	1 (7.69)	1 (10.00)	2 (15.38)	1 (10.00)	0 (0.00)	1 (10.00)	2 (15.38)	3 (30.00)	5 (38.46)	3 (30.00)

* p<0.05,

** p<0.01 statistically significant

Legend: P0: before surgery; P1: 3 months after surgery; P2: 6 months after surgery TG: treated group; UTG: untreated group; CG: control group

Statistical tests used: Comparison between periods and comparison with control: Fisher exact test

Table 4 presents the results regarding the electromyographic activity of the masseter and temporalis muscles, in TG and UTG for each study period. Comparing the groups in P1, the EMG of RM, RT was lower for TG than the respective CG.

**Table 4:** Means and standard deviation of the percentage of muscle activity for the TG, UTG and CG in the evaluation periods

		RM		LM		RT		LT
	TG	UTG	TG	UTG	TG	UTG	TG	UTG
P0	71.57	93.96	88.17	69.8	77.52	65.76	67.57	57.08
	±33.18	±57.66	±51.41	±41.04	±33.09	±15.86	±28.95	±23.66
P1	64.94	77.93	77.2	84.98	58.84	83.51	75.75	72.83
	±21.39	±30.99	±36.62	±24.37	±16.51	±46.12	±54.08	±24.13
P2	75.14*	92.54	79.6	88.7	64.1*	64.57	66.91	67.2
	±36.37	±33.14	±35.73	±29.24	±17.46	±23.72	±23.07	±25.66
CGα	103.8	75.89	102.06	71.4	83.8	58	86.72	70.56
	±40.01	±22.96	±42.33	±22.87	±20.89	±23.32	±24.52	±36.20

* p<0.05 statistically significant

P0: before surgery; P1: 3 months after surgery; P2: 6 months after surgery; TG: treated group; UTG: untreated group; CG: control group

RM: right masseter; LM: left masseter; RT: right temporalis; LT: left temporalis

αCG was not treated

Statistical tests used: Comparison between periods Anova/Tukey or Friedman/Dunn; Comparison with control: Anova/Dunnet

The results concerning duration of the masticatory act and cycle of each muscle are presented in Table 5. In TG the RM muscle showed lower values in P2 than P0 and P1. The EMG values of RM at P0 were higher than the CG for both groups, whereas for LM at P0 only UTG showed higher values compared with their controls. At P1, only TG presented higher values than CG for RM. The results related to duration of the masticatory cycle. The values for RM in TG at P2 were significantly lower than P0 and P1. The values for RM and RT at P0 for TG and UTG were higher than CG.

**Table 5:** Means and standard deviation of the act and cycle duration for the TG, UTG and CG in the evaluation periods between TG and UTG

				Act duration								cycle duration				
	RM		LM		RT			LT	RM		LM		RT			LT
	TG	UTG	TG	UTG	TG	UTG	TG	UTG	TG	UTG	TG	UTG	TG	UTG	TG	UTG
P0	0.37	0.41	0.34	0.39	0.29	0.31	0.29	0.35	0.84	0.89	0.84	0.89	0.9	1.01	0.86	0.84
	±0.11	±0.12	±0.14	±0.09	±0.08	±0.09	±0.09	±0.30	±0.18	±0.17	±0.25	±0.30	±0.23	±0.35	±0.21	±0.21
P1*	0.35	0.32	0.33	0.32	0.29	0.29	0.3	0.3	0.81	0.74	0.8	0.71	0.84	0.79	0.83	0.74
	*±0.10	*±0.05	*±0.11	±0.05*	±0.12	±0.09	±0.10	±0.09*	*±0.26	±0.08*	±0.26	±0.19	±0.26*	±0.14*	±0.26	±0.06
P2	0.29*	0.32	0.28	0.3	0.23	0.27	0.25	0.26	0.68	0.77	0.7	0.74	0.72	0.78	0.74	0.78
	±0.05	±0.08	±0.05	±0.06	±0.06	±0.06	±0.05	±0.04	±0.15	±0.12	±0.14	±0.06	±0.16	±0.09	±0.20	±0.06
CGα	0.26	0.26	0.26	0.27	0.25	0.24	0.24	0.23	0.74	0.74	0.72	0.74	0.74	0.76	0.74	0.75
	±0.05	±0.04	±0.04	±0.06	±0.04	±0.04	±0.04	±0.04	±0.09	±0.09	±0.09	±0.09	±0.08	±0.12	±0.07	±0.13

* p<0.05 statistically significant

P0: before surgery; P1: 3 months after surgery; P2: 6 months after surgery; TG: treated group; UTG: untreated group; CG: control group

RM: right masseter; LM: left masseter; RT: right temporalis; LT: left temporalis

αCG was not treated

Statistical tests used: Comparison between periods: Anova/Tukey or Friedman/Dunn; Comparison with control: Anova/Dunnet

Table 6 contains the values of the TG and UTG on the number of chewing cycles in different periods. At P2 the TG showed more cycles than in P0. Comparing the groups before surgery, the UTG showed fewer masticatory cycles than the CG.

**Table 6:** Means and standard deviation of the number of cycles for the TG, UTG and CG in the evaluation periods

Number of cycles		
	TG	UTG
P0	11.34	10.53
	±2.87	±1.82
P1	*12.04	12.63
	±2.45	±1.16*
P2	13.79	12.6
	±2.44	±1.24
CGα	12.55	12.6
	±1.42	±1.87

* p<0.05 statistically significant

P0: before surgery; P1: 3 months after surgery; P2: 6 months after surgery; TG: treated group; UTG: untreated group; CG: control group

RM: right masseter; LM: left masseter; RT: right temporalis; LT: left temporalis

αCG was not treated

Statistical tests used: Comparison between periods: Anova/Tukey; Comparison with control: Anova/Dunnet

**Figure 4:**
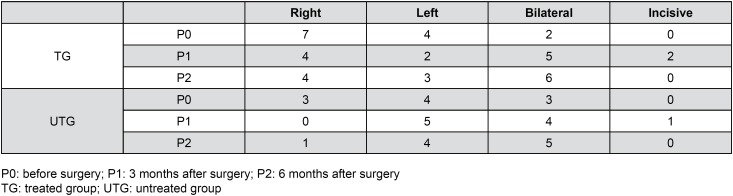
Individuals classified according to the chewing side preference

The distribution of individuals according to the chewing side preference is shown in Figure 4.

## Discussion

Besides esthetic and morphological problems, individuals with DFD may present alterations in stomatognathic functions, particularly in masticatory muscle activity. Morphological and functional analysis are important for diagnosis and evaluation of treatment outcomes^21^ ^,^ ^26^. Thus, the clinical and instrumental aspects of masticatory function in individuals undergoing OGS, as well as the effect of orofacial myofunctional therapy were verified.

TG presented increase in maximum scores of OMES-E three and six months after surgery compared to the preoperative period, indicating improvement in masticatory function and the effect of OMT. Similar results were not observed in UTG. Pereira and Bianchini^18^ (2011) also observed improvement in masticatory function four months after OGS in patients with Class II malocclusion submitted to orofacial myofunctional therapy.

The maximum scores of the TG and UTG differed from their controls in all periods, showing that in the TG, although there was improvement six months after OGS, the values still did not approach the pattern of control individuals. This finding agreed with Van den Braber, et al.^27^ (2006), who observed improvement in masticatory performance five years after OGS, but the function was still impaired when compared with controls.

In the analysis of each item in OMES-E protocol in relation to the “masticatory type” before surgery, both sub-groups presented alteration in this aspect compared with the control. A clinical evaluation of masticatory function in individuals with DFD also found changes in the mastication type^17^. In the present study, six months after surgery, the TG showed significant increase in scores of mastication type, suggesting improvement in function, and these results were not observed in UTG. However, comparing the TG and UTG with their counterparts, after surgery, the scores of the TG were significantly lower than the CG; therefore, despite the improvement, the values did not approach the control. In relation to the item “bite”, the scores for TG and UTG were similar to their controls at P0. Moreover, no significant differences were observed after surgery, showing that the DFD did not interfere with this aspect.

At P0, four individuals of the TG showed alteration in head movements during chewing, whereas in CG none was altered, as expected. In UTG two individuals showed the respective alteration. A direct functional relation between the head and neck posture was observed during chewing^20^, and possible changes that could interfere with it, such as muscles and mandibular posture, could explain the alteration found in those individuals. At P1 only UTG differed from CG, showing an improvement in the TG, since only one individual showed alteration in this aspect. Over time, there was great variability in this item that could be attributed to individual variation at the moment of evaluation and also to the subjectivity of the test. Thus, it was not possible to confirm the effect of OMT for head movements over the six months after surgery.

Only one individual of TG showed alteration in head posture at P1; nonetheless, recovering was observed at P2, since the number of individuals with alteration was similar to P0. Despite this, no significant differences were found between periods. It should be considered that UTG differed from its control at P0 and P2, whereas TG was close to CG with more individuals without alteration. It has been asserted that changes in occlusion can influence the muscular balance and head position^17^. Some studies have found forward head posture, especially in individuals with Class II malocclusion^10^. In the UTG there were more individuals with Class II malocclusion, which may have contributed to the fact that before surgery more individuals of this group showed alteration in head posture during chewing.

Food escape was evaluated and it should be considered that, before surgery, the DFD did not influence this aspect, since the values were similar to the control; no difference was observed after surgery. The literature shows that many patients experience paresthesia after orthognathic surgery, mainly at the lips and chin^12^; thus, food escape could be expected, although it did not occur. However, the first evaluation occurred 3 months after surgery and this period may suffice for adjustment of this aspect.

The lower lip tone before surgery for TG was altered in all individuals and in UTG only one individual presented normality. Similarly to this finding, a study showed reduced tone of the elevator muscles of the jaw, buccinator muscles and lips in individuals with DFD^1^. Three and six months after surgery, in the TG, there was improvement compared to the preoperative period and the number of individuals was close to the control, showing improvement in this aspect; the same was not observed in the UTG. Therefore, there was effect of therapy in relation to the lower lip tone. In relation to tone of the tongue, even after surgery, the values were different from the control, showing that six months were not sufficient for tongue adaptation.

After surgery, more individuals of TG presented adequate lip mobility, but no differences were found between periods, perhaps due to the small number of subjects. TG presented higher number of individuals with adequate tongue mobility three and six months after surgery compared to the preoperative period, and after surgery only the UTG differed from the control. Therefore, it could suggest that the OMT contributed to improve muscle mobility. To our knowledge, no studies could be found that describe this aspect in patients undergoing OGS, evidencing the importance of the findings and emphasizing that mobility should be evaluated and treated during OMT.

Regarding EMG of masticatory muscles, the data for muscle activity were analyzed in different periods and no significant difference was found after OGS. After three months TG presented significantly lower EMG values than CG for the right masseter and right temporalis. The UTG did not show similar differences. These findings can suggest that the OMT has little influence on EMG, probably due to the evaluation periods after surgery. Thus, the time needed to obtain improvement of EMG activity after orthognathic surgery can be considered a controversial issue. Some studies found no difference over a period of one year^13^ ^,^ ^14^, while others showed increase in EMG activity while chewing, six months^22^ and three years^23^ after surgery compared with the preoperative period. Moreover, in the present study, even before surgery the EMG values of the experimental groups were not different from the controls, probably due to the previous functional adaptation to the abnormal anatomic structures. The variability of EMG data can be a contributing factor, despite the care in signal acquisition, plus the surroundings factors, including muscle length, muscle anatomy, electrode position and characteristics of contraction filaments^5^, which could influence the EMG results about the effect of OMT on EMG data.

The duration of the masticatory act and cycle for the RM decreased significantly in TG over the six months after OGS, suggesting that the individuals began to perform chewing cycles with shorter duration, including the occlusal phase. Despite a possible adaptation to malocclusion in individuals with DFD, as mentioned above, the abnormalities present before surgery could be damaging the masticatory efficiency due to muscle imbalance, increasing cycle duration to improve mastication. After surgery, the reestablishment of dentofacial balance added to OMT may have improved the masticatory efficiency. These findings corroborate the results found by Kobayashi, et al.^16^ (2001), who analyzed patients with Class III malocclusion and found a reduction in the masticatory rhythm in the postoperative period compared to the preoperative. Conversely, other studies found no change in this aspect after OGS^13^ ^,^ ^26^.

The results confirmed the effect of treatment on the right masseter muscle. In this context, it can be observed that the side of masticatory preference of TG was predominantly the right side mainly in P0, which is in line with masticatory preference side in TG, since the right side was predominant at P0, and present at P1 and P2. The difficulty in maximum intercuspation in Class II malocclusion associated with mandibular movement during chewing can determine functional adaptations, such as unilateral chewing to facilitate the process^18^. Thus, the presence of individuals with Class II malocclusion may have influenced the unilateral pattern.

The experimental groups showed significantly longer cycle duration in RM and RT at P0 than CG. This probably occurred to compensate dental-occlusal and muscle disorders. According to Engelen, et al.^6^ (2005), individuals with impaired masticatory performance often compensate it by a higher number of chewing cycles, resulting in longer duration of masseter muscle activity. For act duration, three months after surgery, only the TG differed from the control for RM, showing that TG was different from the control. Thus, it is possible to consider that three months were not enough to detect positive results of OMT. However, six months after OGS, the groups approached the control with better results than P1 and P0. During the therapy sessions, masticatory function was exercised using latex rubber and natural foods in order to promote balance of this function, reflecting an improvement on the occlusal phase and cycle duration.

The present study did not find differences in muscle activity, but the improvement observed in masticatory duration six months after surgery can suggest that the effect of treatment remained until this time. The EMG results differ from Ko, et al.^15^ (2015), who observed that individuals with Class III malocclusion undergoing physical therapy after OGC, consisting of active and passive jaw exercises and dietary instruction, showed greater EMG of the masseter and temporalis muscles in relation to the untreated group after six weeks. Nevertheless, after six months no difference between groups was detected.

An increase was observed in the number of chewing cycles six months after surgery in the TG, explained as the result of lower cycle duration, and consequently more cycles were performed. Corroborating these results, a recent research showed increasing trend of the total number of chewing cycles after 36 months of orthodontic-surgical treatment in patients with Class III malocclusion, determining improvement in the balance of masticatory muscles after surgery^19^.

Therefore, the OMT brought favorable physiological changes in the performance of electromyographic duration with decrease in act and cycle and increase in the number of chewing cycles after surgery. Furthermore, the clinical results showed that the orofacial myofunctional therapy could provide improvement in aspects related to maximum score of OMES-E, masticatory type, lower lip tone and tongue mobility. It was not possible to prove the enhancement in all items of the OMES-E protocol, considering that chewing is a complex physiological function involving neuromuscular activities^12^ and individual's behavior and attitudes^11^.

Many studies discuss the results about the functional characteristics of masticatory muscles in individuals with DFD undergoing OGS^9^ ^,^ ^15^ ^,^ ^23^ but few studies have been conducted considering the objective and subjective chewing aspects^18^ ^,^ ^22^. Thus, the present study contributes to these findings, stressing the importance of evaluation and myofunctional therapy in cases of OGS. Similar studies should be conducted with greater number of individuals, and addressing other orofacial functions.

## Conclusion

The effect of treatment was observed in clinical and electromyography aspects. Thus, the importance of OMT for individuals with DFD undergoing OGS becomes evident.
